# Non-stationary component extraction in noisy multicomponent signal using polynomial chirping Fourier transform

**DOI:** 10.1186/s40064-016-2849-2

**Published:** 2016-07-26

**Authors:** Wenlong Lu, Junwei Xie, Heming Wang, Chuan Sheng

**Affiliations:** Air and Missile Defense School, Air Force Engineering University, 710051 Xi’an, China

**Keywords:** Polynomial chirping Fourier transform, Particle swarm optimization, Component extraction, Noisy multicomponent signal

## Abstract

Inspired by track-before-detection technology in radar, a novel time–frequency transform, namely polynomial chirping Fourier transform (PCFT), is exploited to extract components from noisy multicomponent signal. The PCFT combines advantages of Fourier transform and polynomial chirplet transform to accumulate component energy along a polynomial chirping curve in the time–frequency plane. The particle swarm optimization algorithm is employed to search optimal polynomial parameters with which the PCFT will achieve a most concentrated energy ridge in the time–frequency plane for the target component. The component can be well separated in the polynomial chirping Fourier domain with a narrow-band filter and then reconstructed by inverse PCFT. Furthermore, an iterative procedure, involving parameter estimation, PCFT, filtering and recovery, is introduced to extract components from a noisy multicomponent signal successively. The Simulations and experiments show that the proposed method has better performance in component extraction from noisy multicomponent signal as well as provides more time–frequency details about the analyzed signal than conventional methods.

## Background

Component extraction plays an important role in multicomponent signal applications, e.g., rotary machine (Pineda-Sanchez et al. 2011), power system (González et al. 2008), radar and sonar (Yeary et al. 2012; Zeng et al. 2010), speech (Pertila and Nikunen 2015), wind turbine (Yang et al. 2009), biomedicine (Motamedi-Fakhr et al. 2014). However, multicomponent signals from a noisy environment, particularly radar signals in complicated battlefield, are typically too heavily interfered to separate.

Many efforts have been devoted to these multicomponent non-stationary signals, mainly based on signal decomposition and adaptive time–frequency methods. Among the signal decomposition methods, Hilbert Huang Transform (HHT) employs empirical mode decomposition (EMD) to express the multicomponent signal with a group of intrinsic mode functions (IMF) (Huang et al. 1998). Each IMF is treated as an amplitude-frequency modulated signal, whereas not all IMFs have physical meaning. Moreover, EMD is typically confronted with problems of mode aliasing and end effect. Ensemble Empirical Mode Decomposition is proposed in (Wu and Huang 2009) to solve the mode aliasing problem of EMD. However, computational complexity will be largely increased. Another effective method is based on energy separation and Gabor filtering (Maragos et al. 1993) but distortion will be introduced in the separated components. To rectify the drawback, a Fourier-Bessel (FB) expansion-based method (Pachori and Sircar 2010) is exploited to extract components from a multicomponent AM-FM signal. Nevertheless, manual determination of the range of FB coefficients means prior information needed and may be a limitation for its application. Besides, an iterative approach is proposed (Jainn and Pachori 2015) based on eigenvalue decomposition of Hankel matrix and mono-component signal criteria. The method decomposes a multicomponent non-stationary signal into narrow-band AM-FM components, whereas the effect will be discounted for wide-band components and crossed components.

As the oldest member of spectrum analysis, Fourier transform (FT) and band-pass filter are the most commonly used method to extract components from a stationary or non-stationary signal based on their separable frequency bins (Boashash 1992). However, the FT-based method fails due to unavailable central frequency and bandwidth of filter when components are submerged in strong noise or overlapped in frequency domain. One improved method is to employ an adaptive time–frequency filter whose central frequency and bandwidth are determined by a local instantaneous frequency (IF) (Lee 2011). Time–frequency methods are typically performed by two approaches: non-parameterized time–frequency analysis (NPTFA) and parameterized time–frequency analysis (PTFA); the former is defined without signal-dependent parameters, and the latter has signal-dependent parameters. Several non-parameterized time–frequency transforms are introduced to achieve local IFs, e.g., Short-Time Fourier Transform (STFT) (Kwok and Jones 2000), Wavelet Transform (WT) (Chen et al. 2016) as well as Wigner-Ville Distribution (WVD) (Hussain and Boashash 2002). These transforms achieve local IFs and update the parameters of adaptive filter. However, they suffer from either poor time–frequency concentration or interference of cross-terms in dealing with multicomponent signals. The matter is even worse in noisy environment. Comparatively, parameterized methods adopt an extra parameterized kernel correlated with the frequency modulated (FM) information of the analyzed signal to approach its time–frequency energy ridge The typical parameterized transforms include Chirplet Transform (CT) (Cui and Wong 2006), polynomial chirplet transform (PCT) (Yang et al. 2013), Spline Chirplet Transform (SCT) (Yang et al. 2012), Warblet Transform (WBT) (Angrisani et al. 2005) and generalized warblet transform (GWBT) (Yang et al. 2012). These parameterized transforms can continuously converge to the IF of the analyzed signal by an iterative procedure involving transform, ridge extraction and parameter updating, though the iteration will divergent because of their blurred ridges in strong noise.

From a perspective of energy accumulation, FT can largely improve signal-to-noise ratio (SNR) because of its energy collection in the whole time domain. The PCT can approach the true time–frequency feature of the analyzed signal because of its energy accumulation of the windowed signal in an optimal path. Inspired by track-before-detection (TBD) (Deng et al. 2011) technology in radar, a polynomial chirping Fourier transform (PCFT) is exploited to integrate advantages of above two transforms. In the PCFT, the time–frequency beelines are replaced with a family of polynomial chirping curves to collect signal energy. Particle swarm optimization (PSO) (Schutte et al. 2004) is utilized to search optimal polynomial parameters that enable the PCFT to obtain most concentrated energy spectrum for the target component. Then a narrow-band filter and the inverse PCFT are employed to extract and reconstruct the component, respectively. In the end, a recursive procedure based on PCFT is addressed for successive component extraction from the noisy multicomponent signal. Simulations and experiments indicate that the proposed method performs better than conventional global transforms and parameterized methods.

The special contributions of this study include:

1. The conventional global transforms and parameterized methods are reinterpreted from a perspective of energy accumulation.
2. According to the new perspective, a novel global transform, i.e., PCFT, is proposed to collect signal energy in a nonlinear way for an improved SNR in strong noise.
3. PSO is utilized to search optimal polynomial parameters by converting the selection of polynomial curves to a nonlinear parameter optimization based on spectrum concentration principle.
4. A recursive procedure based on PCFT is addressed for sequential component extraction from a noisy multicomponent signal.

The remainder of this paper is organized as follows. “Theoretical background” section reviews the theoretical background concerning conventional time–frequency transforms. “The proposed method” section describes the PCFT-based method for component extraction. “Simulations and experiments” section evaluates the studied method on several numerical and experimental examples. Finally, some conclusions are drawn in final section.

## Theoretical background

### Noisy multicomponent signal

A noisy multicomponent FM signal can be expressed as the sum of sinusoidal functions and noise, which is defined as (Yang et al. 2014)

1 $$ s(t) = \sum\limits_{k = 1}^{M} {s_{k} (t) + n(t)} $$

where $n\left( t \right)$ is white Gaussian noise of power $\sigma^{2}$ and mean of 0. The SNR (Wang et al. 2015) is defined as

2 $$ SNR = 10\log_{10} \left( {{{P_{s} } \mathord{\left/ {\vphantom {{P_{s} } {P_{n} }}} \right. \kern-0pt} {P_{n} }}} \right) $$

where $P_{s}$ and $P_{n}$ are the energy of multicomponent signal and noise. *M* is the number of the components. The analytic formulation of the *k*th component $s_{k} (t)$, $s_{k} \left( t \right) \in L^{2} \left( R \right)$, can be achieved by Hilbert transform as follows.

3 $$ z_{s,k} (t) = s_{k} (t) + j{\text{H}}[s_{k} (t)] $$

where the Hilbert transform of $s_{k} (t)$ is

4 $$ {\text{H}}[s_{k} (t)] = \frac{1}{\pi }P.\,V.\int_{ - \infty }^{ + \infty } {\frac{{s_{k} (\tau )}}{t - \tau }d\tau } . $$

*P. V.* means the integral taken in the sense of Cauchy principal value.

The analytic formulation of the analyzed signal can also be rewritten as (Stankovic et al. 2014)

5 $$ z(t) = \sum\limits_{k = 1}^{M} {A_{k} (t)exp\left[ {j\phi_{k} (t)} \right]} + z_{n} \left( t \right) $$

where every IF is $f_{k} = {{\phi^{\prime}_{k} \left( t \right)} \mathord{\left/ {\vphantom {{\phi^{\prime}_{k} \left( t \right)} {2\pi }}} \right. \kern-0pt} {2\pi }}$. As $A_{k} \left( t \right)$ varies slowly compared with the IF of the component, it is typically considered as a constant amplitude. If *M* = 1, the signal degenerates into a monocomponent signal. The model defined by (1)–(5) applies for noisy multicomponent signal and allows the modeling of *M* time–varying frequency laws.

### FT

The FT and Inverse FT are an effective tool to analyze stationary signal. The transforms are defined as follows.

6 $$ \left\{ \begin{aligned} &S\left( \omega \right) = \frac{1}{2\pi }\int_{ - \infty }^{ + \infty } {z\left( \tau \right)} \exp \left( { - j\omega \tau } \right)d\tau \hfill \\ &z\left( t \right) = \int_{ - \infty }^{ + \infty } {S\left( \omega \right)} \exp \left( {j\omega \tau } \right)d\omega \hfill \\ \end{aligned} \right. $$

FT can be considered as the energy accumulation of the analyzed signal along a time–frequency beeline parallel to time axes. The global transform converts the analyzed signal from time domain into spectral representation. The energy-concentrated spectrum largely improves SNR for a narrow-band signal, particularly for the monochromatic signal. Then a band-pass filter can be utilized to separate the concentrated component in frequency domain.

**Fig. 1:**
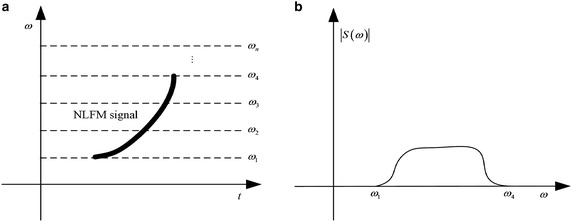
TFR and spectrum of a NLFM signal. **a** TFR. **b** Signal spectrum

However, FT-based method are not suitable for spectrum-crossed components, where the matter is even worse in noise environment. Figure 1 shows a time–frequency representation (TFR) and spectrum of a nonlinear frequency modulated (NLFM) signal. It can be learned that the IF of the non-stationary wideband signal varies with time. The signal energy is distributed broadly in spectrum. In this case, the transform projects each piece of the energy of the signalinto spectrum but accumulate noise in the whole time domain, leading to difficult signal dectection.

### STFT and PCT

The STFT is an extension of the FT for non-stationary signal analysis. The STFT divides a non-stationary signal into time pieces with a window function. Each piece is assumed as stationary and processed with FT.

7 $$ STFT\left( {t,\omega } \right) = \int {z\left( \tau \right)h\left( {t - \tau } \right)\exp \left( { - j\omega \tau } \right)} d\tau $$

where $h\left( t \right)$ is the window function. The transform obtains the local IF of the analyzed signal by mapping the one-dimensional signal into a two-dimensional function of time and frequency. Unfortunately, STFT is limited for better concentration due to its window effect where a tradeoff must be made between time and frequency resolution.

A modified window function $h\left( t \right)$ is employed in PCT to improve time–frequency resolution. As an alternative, the PCT is a generalization of the STFT with supplementary degrees of freedom for the window function.

8 $$ PCT(t,\omega ;c_{2} ,c_{3} , \ldots ,c_{n} ,\sigma ) = \int_{ - \infty }^{ + \infty } {z(\tau )\psi (\tau ,t;c_{2} ,c_{3} , \ldots ,c_{n} ,\sigma )} \exp ( - j\omega \tau )d\tau $$

where $\psi \left( {\tau ,t;c_{2} ,c_{3} , \ldots ,c_{n} ,\sigma } \right)$ is a modified window given as

9 $$ \psi (\tau ,t;c_{2} ,c_{3} , \ldots ,c_{n} ,\sigma ) = h_{\sigma } (\tau - t)\exp \left[ { - j\sum\limits_{i = 2}^{n} {\frac{{c_{i} }}{i}(\tau - t)^{i} } } \right] $$

where *t* and $\left( {c_{2} ,c_{3} , \ldots ,c_{n} } \right) \in R$ are time and polynomial chirping cofficients. $h_{\sigma } \in L^{2} \left( R \right)$ indicates a window function which is typically taken as a Gaussian function.

10 $$ h_{\sigma } (t) = \frac{1}{{\sqrt {2\pi } }}\sigma \exp \left[ { - \frac{1}{2}\left( {\frac{t}{\sigma }} \right)^{2} } \right] $$

where $\sigma$ determines the length of the Gaussian window.

By using polynomial chirplet, the PCT shapes the window with more degrees of freedom, i.e., it can not only shear and scale the window along the time and frequency axes, but also bend and rotate eah cell in the time–frequency plane. New degrees of freedom in shaping the window cells mean better energy collection mode for PCT to concentrate the energy ridge of the analyzed signal in the time–frequency plane. The PCT extends its ability to analyze NLFM signals because the modified window covers more energy within the same time–frequency region.

The PCT is a iterative ridge-extraction method. The coefficients of the PCT can be obtained by approaching the energy ridge of the analyzed signal with a polynomial function. The STFT provides the initial time–frequency energy ridge for the iterative procedure. Then the PCT is performed with estimated parameters to obtain another TFR. The PCT continuously converges to the IF of the analyzed signal with a parameterized kernel by a recursion involving transform, ridge extraction and parameter estimation. However, the ridge-extraction method is overwhelmed when useful components are submerged in strong noise. The energy ridge within a short-duration constant window becomes smeared in noise, leading to divergence of the iteration.

## The proposed method

### PCFT

The PCT of the signal is considered in first window piece. According to definitions in (8)–(10), the $PCT(0,\omega ;c_{2} ,c_{3} , \ldots ,c_{n} ,\sigma )$ can be considered as the energy collection of the windowed signal along a polynomial chirping curve. The $PCT(0,\omega ;c_{2} ,c_{3} , \ldots ,c_{n} ,\sigma )$ of the signal can be rewritten as

11 $$ PCT(0,\tilde{\omega };c_{2} ,c_{3} , \ldots ,c_{n} ,\sigma ) = \int_{ - \infty }^{ + \infty } {z_{h} (\tau )\exp \left( { - j\tilde{\omega }\tau } \right)} d\tau $$

with

12 $$ z_{h} \left( \tau \right) = z\left( \tau \right)h_{\sigma } \left( \tau \right) $$

13 $$ \tilde{\omega } = \omega + \sum\limits_{i = 2}^{n} {\frac{{c_{i} }}{i}\tau^{i - 1} } $$

where $\tilde{\omega }$ is defiend as a new angular frequency curve which is represented as a polynomial chirping curve in the time–frequency plane. And the transform congregates energy of the windowed signal $z_{h} \left( \tau \right)$ along the curve. When $\tilde{\omega }$ approaches the IF of $z_{h} \left( \tau \right)$, $PCT(0,\omega ;c_{2} ,c_{3} , \ldots ,c_{n} ,\sigma )$ will obtain increasingly concentrated energy ridge. Therefore, the PCT can approximate the IF of the analyzed signal with a ridge-extraction method. However, the method fails in noisy environment due to limited energy of the signal within the short-duration window.

The problem in PCT is similar to weak target detection in radar. The conventional target detection employs the same technology in radar, i.e., detection and track, where signal detection is a precondition of track. In a low-SNR environment, extracted ridge by peak location algorithm can not reveal the time–frequency feature of the analyzed signal any longer. Therefore, parameter estimation fails when component energy is submerged in strong noise.

Inspired by TBD technology, the window function in the PCT can be removed to convert the local transform to be a global one, i.e., PCFT, which can acquire the same advantage as FT in signal energy accumulation. The PCFT transforms the analyzed signal from the time domain into a polynomial chirping Fourier domain, where the analyzed signal obtains a concentrated energy-concentrated spectrum and maximize energy peak with optimal parameters $\left( {c_{2} ,c_{3} , \ldots ,c_{n} } \right)$. To be simple, the angular frequency curve $\tilde{\omega }$ is rewritten as

14 $$ \tilde{\omega } = \omega + \sum\limits_{i = 1}^{n} {\alpha_{i} t^{i} } , $$

where $\left( {\alpha_{1} ,\alpha_{2} , \ldots ,\alpha_{n} } \right)$ are new polynomial parameters and *n* represents the order of polynomial chirping. As is known, the utilization of high order polynomial can improve IF approximation as well as lead to Runge phenomenon. Therefore, $n \le 3$ in most applications. Like FT, the PCFT and its inverse transform can be defined as follows.

15 $$ \left\{ \begin{aligned} &S(\omega ) = \int_{ - \infty }^{ + \infty } {z(t)\exp \left[ { - j\left( {\omega + \sum\limits_{i = 1}^{n} {\alpha_{i} t^{i} } } \right)t} \right]dt,} \hfill \\ &z(t) = \frac{1}{2\pi }\int_{ - \infty }^{ + \infty } {S(\omega )\exp \left[ {j\left( {\omega + \sum\limits_{i = 1}^{n} {\alpha_{i} t^{i} } } \right)t} \right]d\omega .} \hfill \\ \end{aligned} \right. $$

**Fig. 2:**
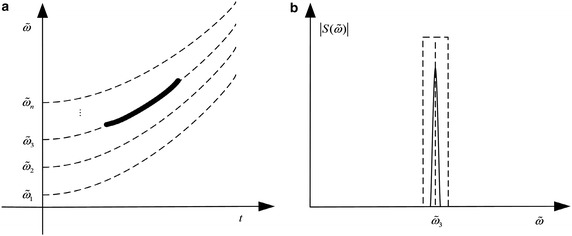
TFR and PCFT spectrum of a NLFM signal. **a** TFR. **b** PCFT spectrum

It can be learned from Fig. 2a that the angular frequency curve in FT is replaced with a parameterized polynomial chirping curve. By the revision, the PCFT collects signal energy along polynomial time–freuqency curves as well as time–frequency beelines. With appropriate polynomial parameters, a NLFM signal can obtain an energy-concentrated polynomial chirping spectrum. In an ideal case, the signal energy is concentrated along the polynomial chirping curve while noise are still distributed in the whole polynomial chirping Fourier domain, as shown in Fig. 2b.

The PCFT is proposed to pick up weak components under heavy noise based on the advantages of FT and PCT. As a global transform, FT can avoid ridge extraction problem in low-SNR environment. As a parameterized method, PCT provides an energy-concentrated time–frequency ridge by optimizing the way of signal accumulation. It is these two merits that the PCFT integrates to obtain an energy-concentrated polynomial chirping spectrum utilized in component extraction.

### Parameter estimation

The parameter estimation of the polynomial chirping curve becomes a problem when PCFT extends its ability to nonlinear frequency-modulated components. According to (15), one group of polynomial parameters correspond to one family of polynomial chirping curves in the time–frequency plane. If polynomial chirping curve approaches the IF of the analyzed signal closely, the signal will be concentrated in the polynomial chirping domain and get the maximum energy peak. The problem of selecting curve family can be considered as an optimization problem which is defined as

16 $$ \{ \tilde{\alpha }_{1} ,\tilde{\alpha }_{2} , \ldots ,\tilde{\alpha }_{n} \} = \arg \mathop {\hbox{max} }\limits_{\omega } \left| {S(\omega ;\alpha_{1} ,\alpha_{2} , \ldots ,\alpha_{n} )} \right| $$

where $\left( {\tilde{\alpha }_{1} ,\tilde{\alpha }_{2} , \ldots ,\tilde{\alpha }_{n} } \right)$ are estimated polynomial parameters and $\omega$ is polynomial chirping Fourier frequency in the new transform domain. The optional solutions to solve this nonlinear optimization problem in (16) include genetic algorithm (Janeiro and Ramos 2009), neural network (Krabicka et al. 2011), PSO, etc. PSO is inspired by the behavior of natural animals as well as Bat Algorithm and Cuckoo Search Algorithm. These algorithms search global optimal parameters by an iterative process. All these algorithms can afford to solve the parameter estimation problem. However, in contrast with other optimal methods, the PSO has less computational complexity and converges to the optimal values quickly. Moreover, the PSO are more suitable for the few-parameter application. For high order PCFT, other optimal algorithms, like Bat Algorithm, may be a better choice. In this paper, the PSO is used for the optimization task for its accuracy and simplicity.

Inspired by the social behavior of bird flocking and fish schooling, the PSO employs a location-velocity model and searches the optimal parameters with a parallel stochastic strategy.. The population of particles corresponds to individual number of parameters. In each search, PSO first initializes a swarm of random particles whose number is in the range [20, 40]. The swarm will search their optimal solutions by an iterative process. Assume that the location and velocity of *i*th particle are ${\varvec{\Lambda}}_{i} = \left( {\alpha_{i,1} ,\alpha_{i,2} , \ldots ,\alpha_{i,N} } \right)$ and ${\mathbf{V}}_{i} = \left( {v_{i,1} ,v_{i,2} , \ldots ,v_{i,N} } \right)$, where *N* indicates problem dimensions. In every iteration, particles are updated according to two optimal results. One is individual optimal location ${\mathbf{P}}_{i} = \left( {p_{i,1} ,p_{i,2} , \ldots ,p_{i,N} } \right)$ found by the particles themselves, which corresponds to the individual extremum $q_{i}$. Another is global optimal location ${\mathbf{P}}_{g} = \left( {p_{g,1} ,p_{g,2} , \ldots ,p_{g,N} } \right)$, corresponding to the global extremum $q_{g}$. The iterative expressions are given as follows.

17 $$ \left\{ \begin{aligned} &v_{{_{i,j} }}^{(k)} = wv_{i,j} (t) + c_{1} r_{1} \left[ {p_{i,j} - \alpha_{{_{i,j} }}^{(k - 1)} } \right] + c_{2} r_{2} \left[ {p_{g,j} - \alpha_{{_{i,j} }}^{(k - 1)} } \right], \hfill \\ &\alpha_{i,j}^{(k)} = \alpha_{i,j}^{(k - 1)} + v_{i,j}^{(k)} ,\quad i = 1,2, \ldots ,M;\;j = 1,2, \ldots ,N, \hfill \\ \end{aligned} \right. $$

where *w* is an inertia weight factor, determining the inheriting and exploring abilities of particles in the swarm. The weight factor is typically determined with a constant, linear decreasing or adaptive method. The $c_{1}$ and $c_{2}$ are two positive learning factors which enable every particle to learn both from their own experiences and the global excellent individuals in order to approach the optimal position in the swarm. The learning factors are typically determined within [0, 4], equal to each other and default as 2. $r_{1}$ and $r_{2}$ are random values in [0, 1].

To sum up, the parameter estimation with PSO includes following six steps:

1. Initialize location ${\varvec{\Lambda}}_{i}^{\left( 0 \right)}$ and velocity ${\mathbf{V}}_{i}^{\left( 0 \right)}$ of every particle randomly within predefined range, $i = 1,2, \ldots ,M$;
2. Calculate objective function in (16) for every particle, store individual optimal locations ${\mathbf{P}}_{i}$, and their extremes $q_{i}$ depending on individual particles, and save the global one ${\mathbf{P}}_{g}$ and $q_{g}$ of the whole swarm;
3. Update individual location ${\varvec{\Lambda}}_{i}^{\left( k \right)}$ and velocity ${\mathbf{V}}_{i}^{\left( k \right)}$ of every particle in *k*th iteration according to (17);
4. Compute objective functions in *k*th iteration, renew ${\mathbf{P}}_{i}$ and $q_{i}$ based on individual particles themselves, and update ${\mathbf{P}}_{g}$ and $q_{g}$ according to the whole swarm;
5. If $\left| {q_{g}^{\left( k \right)} - q_{g}^{{\left( {k - 1} \right)}} } \right| < \varepsilon$($\varepsilon$ is a predefined margin of error), stop and output ${\mathbf{P}}_{g}$ and $q_{g}$; else, go to step 3);
6. If $k > K$ ($K$ is a predefined iteration number), stop and output ${\mathbf{P}}_{g}$ and $q_{g}$; else, go to step 3).

### Component extraction

**Fig. 3:**
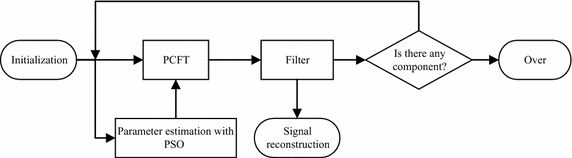
Flowchart of multicomponent signal extraction

Each component of the multicomponent signal is different in their energy. The PSO will approach the strong component by an iterative search. Figure 3 shows the flowchart of multicomponent signal separation. In each component extraction, PSO first approaches the optimal parameters of the strong componet. With the optimal parameters, the PCFT achieves an energy-concentrated spectrum for the component. Then the target component is filtered in polynomial chirping Fourier domain and reconstructed according to (15). The procedure is repeated for the remained signal to extract components successively.

**Fig. 4:**
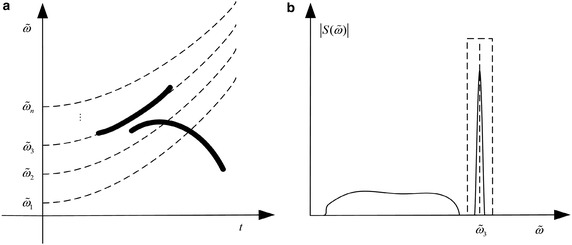
TFR and PCFT spectrum of a signal in case one. **a** TFR. **b** PCFT spectrum

For a multicomponent signal with different IF laws, there are two cases: one is with separable IF laws in the time–frequency plane while the other is with crossed IF laws. As it can be seen in Fig. 4, two components of the analyzed signal are separable in the time–frequency plane as well as in the polynomial chirping Fourier domain. After PCFT, a narrow-band filter can be employed to extract the well-concentrated component.

18 $$ X_{B} \left( \omega \right) = \left\{ \begin{aligned} &X\left( \omega \right),\begin{array}{*{20}c} {} & {\omega_{0} - {B \mathord{\left/ {\vphantom {B 2}} \right. \kern-0pt} 2} < \omega < \omega_{0} + {B \mathord{\left/ {\vphantom {B 2}} \right. \kern-0pt} 2},} \\ \end{array} \hfill \\ &0,\begin{array}{*{20}c} {} & {} & { \, \text{else}} \\ \end{array} , \hfill \\ \end{aligned} \right. $$

where $B$ determines the bandwidth of the filter. Here, the filter is considered as an open-loop adaptive filter whose central frequency varies along with energy peak in order to capture the component. The filtered component $X_{B} \left( \omega \right)$ will be reconstructed with inverse PCFT according to (15).

**Fig. 5:**
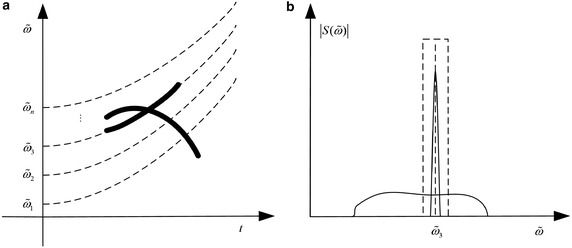
TFR and PCFT spectrum of a signal in case two. **a** TFR. **b** PCFT spectrum

Figure 5 shows the TFR and PCFT spectrum of a multicomponent signal in case two. The strong component of the analyzed signal is concentratedin the transform domain while the other spreads its energy broadly. The component extracted by the filter will necessarily contain partial energy of the other component. The compromise for an appropriate bandwidth should be made between the extracted component and the remain one.

## Simulations and experiments

In this section, a range of examples, including simulated and real-world signals, are utilized to verify the effectiveness of the PCFT-based method for component extraction. Firstly, a simulation study is performed with a monocomponent signal at two SNRs to demonstrate the robustness of the proposed method to noise. Secondly, a nosiy two-component signal is simulated to explain the process of component extraction in noisy environment. Finally, the PCFT-based method is applied to a bat echolocation signal to explore its hidden time–frequency structure. Moreover, several conventional time–frequency methods are performed for comparison.

### Performance with a noisy monocomponent signal

**Fig. 6:**
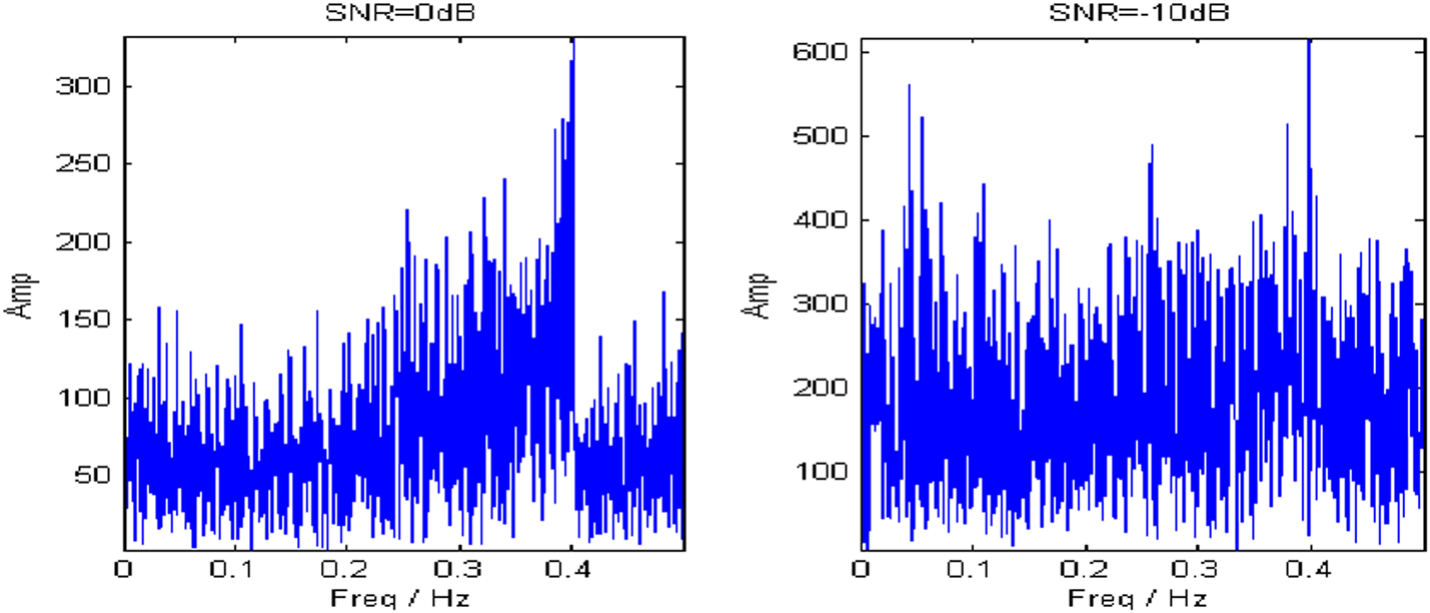
FT spectrums of the signal at two SNRs

In this section, a polynomial phased signal with constant amplitude is given as

19 $$ x\left( t \right) = \sin \left( {1.254t + 1.158 \cdot 10^{ - 3} \cdot t^{2} - 3.517 \cdot 10^{ - 7} \cdot t^{3} } \right) + n\left( t \right) $$

whose the IF is $f\left( t \right) = 0.1996 + 0.3686t + 1.6792t^{2}$. The sampling frequency is normalized and the sampled points are 2000. In order to compare the noise tolerance of the proposed method with other methods, the signal is masked by the white Gaussian noise whose SNRs are 0 and −10 dB, respectively. Figure 6 provides the FT spectrums of the analyzed signal at two SNRs. It can be learned that the noise is too heavy to distinguish from the target component when SNR = −10 dB.

**Fig. 7:**
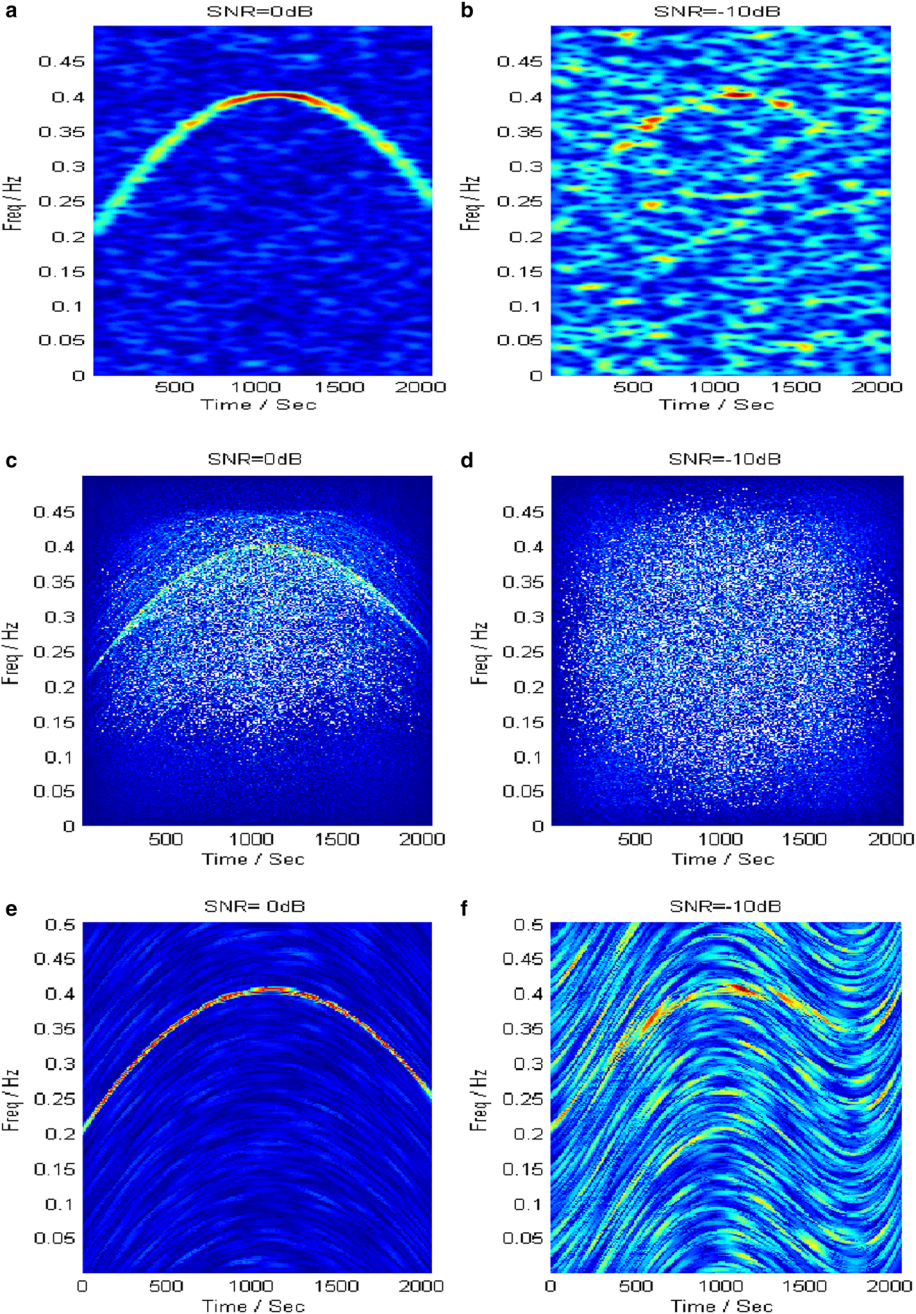
TFRs obtained with different transforms. **a**, **b** STFT. **c**, **d** WVD. **e**, **f** PCT

Figure 7 shows the TFRs generated by STFT, WVD and PCT. Thereinto, the STFT shows poor time–frequency concentration and fails in representing the time–varying IF of the non-stationary signal due to its constant time–frequency resolution. The obtained TFR by STFT at 0 dB only shows an IF silhouette while the IF signature of the signal is totally covered by noise at −10 dB (Fig. 7a, b). The WVD achieves the best time–frequency concentration for the single linear frequency modulated (LFM) signal, yet it is unable to suppress crossed terms. As in Fig. 7c, d, self-crossed terms act as main interference at 0 dB and mutual-crossed terms between the NLFM signal and noise dominate the smeared TFR at the lower SNR. In the PCT, accurate approximation of the IF largely depends on a clear energy ridge in the time–frequency plane, which is not suitable for the low-SNR signal. Therefore, the clear IF at 0 dB becomes smeared in the TFR at −10 dB (Fig. 7e, f).

In contrast with the conventional transforms, PCFT accumulates signal energy in the whole time domain. The parameterized chirping curves of the PCFT are determined by (16) with the PSO algorithm. The population size of PSO is set to 30, and the search regions are set to [−0.1, 0.1] and [−0.01, 0.01] for *α*_1_, *α*_2_ according to sampling frequency. Estimated parameters at both SNRs are obtained by an iterative searche and listed in Table 1.

**Table 1:** Estimated parameters at different SNRs

SNR	$\tilde{a}_{1}$	$\tilde{a}_{2}$
0 dB	1.1571e−3	−3.5155e−7
−10 dB	1.1610e−3	−3.5280e−7

**Fig. 8:**
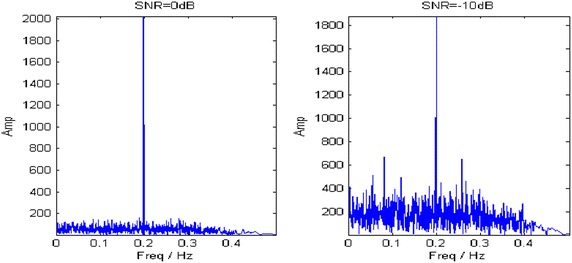
PCFT spectrums at different SNRs

**Fig. 9:**
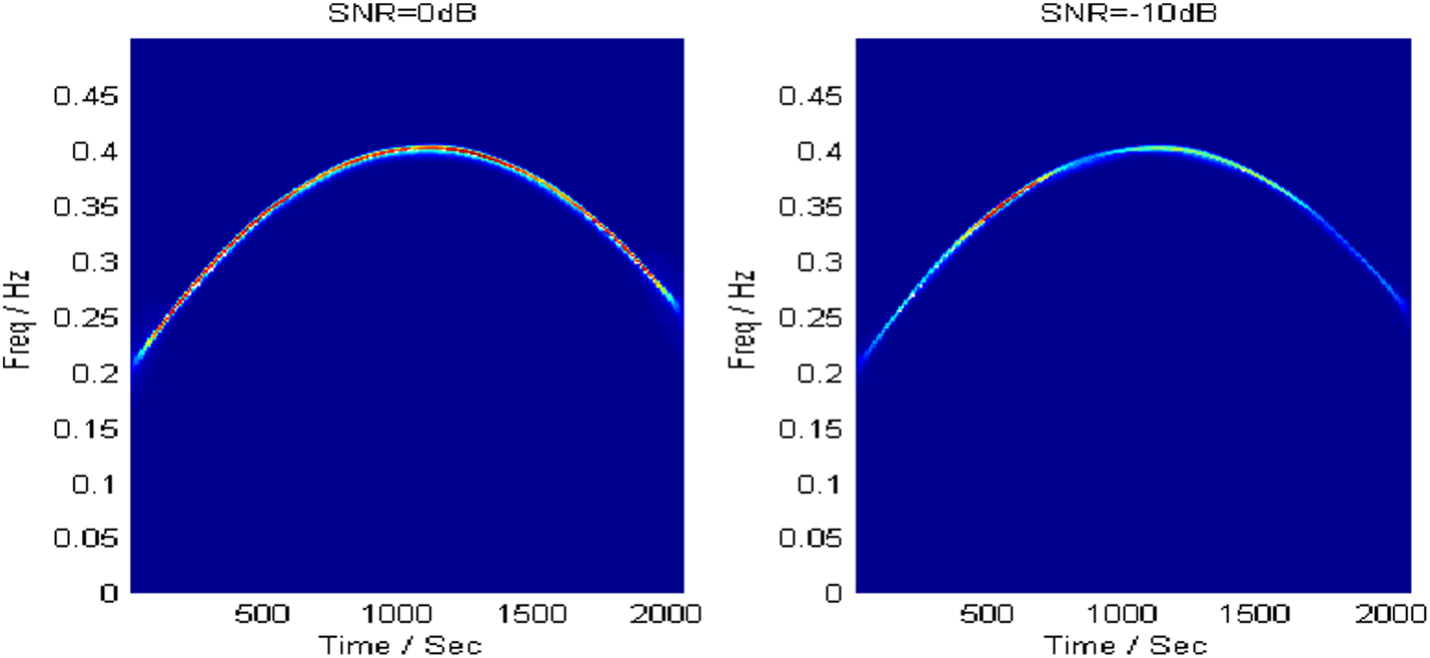
TFRs of the extracted component at different SNRs

With estimated parameters, the component energy is largely concentrated in the polynomial chirping Fourier domain at both SNRs, as shown in Fig. 8. Then the target component is extracted by a band-pass filter with 1 % bandwidth of sampling frequency. Figure 9 describes the TFRs of the extracted component at different SNRs. Both of them accurately characterize the IF of the analyzed signal.

### Performance with a noisy two-component signal

**Fig. 10:**
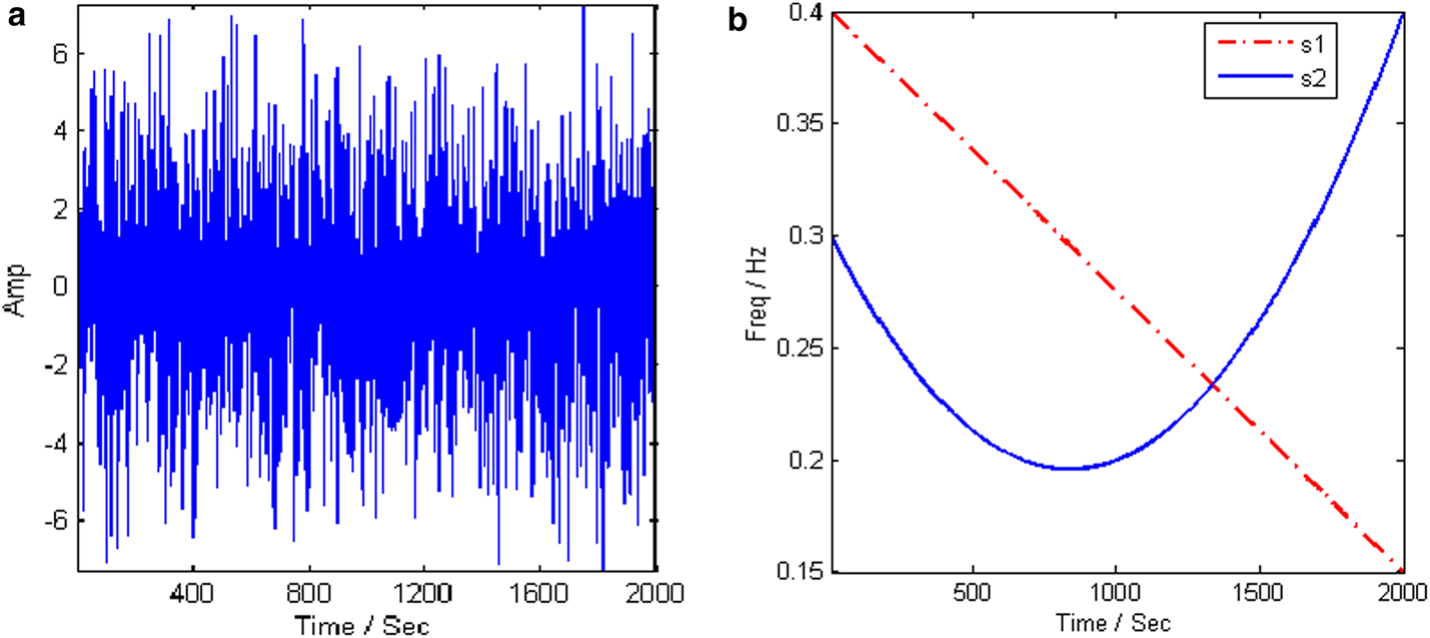
Signal given by (20). **a** Time Waveform. **b** Time–frequency signature

In this subsection, a noisy two-component signal is considered as

20 $$ s\left( t \right) = \kappa_{1} s_{1} \left( t \right) + \kappa_{2} s_{2} \left( t \right) + n\left( t \right) $$

with

21 $$ s_{1} \left( t \right) = \sin \left( {2.513t - 3.927 \cdot 10^{ - 4} \cdot t^{2} } \right) $$

22 $$ s_{2} \left( t \right) = \sin \left( {1.887t - 7.866 \cdot 10^{ - 4} \cdot t^{2} - 3.144 \cdot 10^{ - 7} \cdot t^{3} } \right) $$

whose IFs are $f_{1} \left( t \right) = 0.4 - 1.25 \cdot 10^{ - 4} t$ and $f_{2} = 0.3003 - 2.504 \cdot 10^{ - 4} t + 1.501 \cdot 10^{ - 7} t^{2}$, respectively. The two mixing coefficients are $\kappa_{1} = 0.8$ and $\kappa_{2} = 0.6$. The multicomponent signal is masked by the noise with an SNR of −10 dB. The sampling frequency is normalized and the sampled points are 2000. Figure 10 gives the waveform of the noisy signal and its real time–frequency signature. In the PSO, the size of the population is set to 30, and the search regions are set to [−0.1, 0.1] and [−0.01, 0.01] for *α*_1_, *α*_2_.

**Fig. 11:**
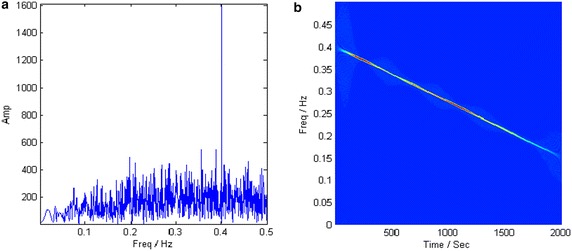
PCFT spectrum and TFR of the first component. **a** PCFT spectrum. **b** TFR

**Fig. 12:**
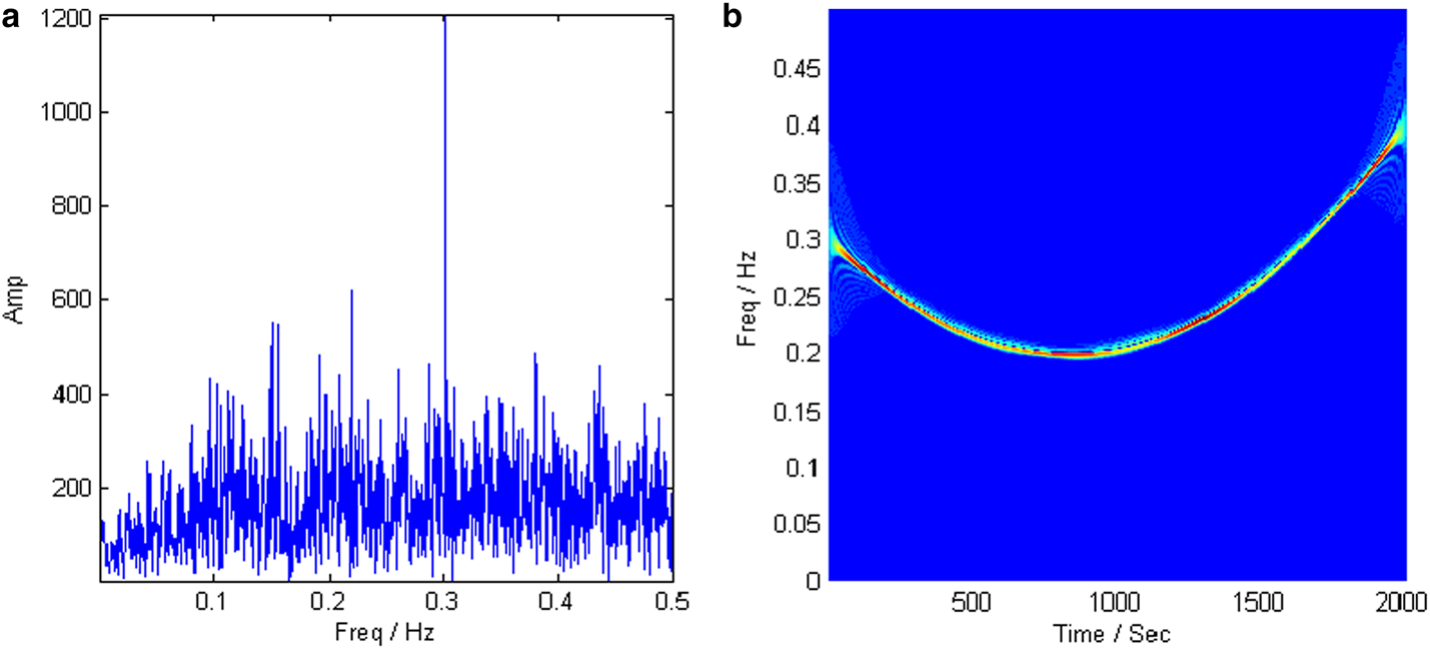
PCFT spectrum and TFR of the second component. **a** PCFT spectrum. **b** TFR

The PCFT firstly approaches the strong component with the estimated parameters obtained by PSO. As seen from the PCFT spectrum of the analyzed signal in Fig. 11a, the amplitude of the matched component is much larger than that of noise and the other component. The target component is extracted from the crossed components by a band-pass filter with 1 % bandwidth of sampling frequency. Figure 11b reveals that the TFR of the first component is a LFM signal. The PCFT-based method is repeated with the remained signal. The PCFT spectrum of the remained signal and the TFR of the extracted component are depicted in Fig. 12. The estimated parameters of two components are listed in Table 2.

**Table 2:** Estimated parameters of two components

Component	$\tilde{a}_{1}$	$\tilde{a}_{2}$
Component 1	−3.9217e−4	−2.2204e−10
Component 2	−7.8475e−4	3.1383e−7

### Application in real-world signal

**Fig. 13:**
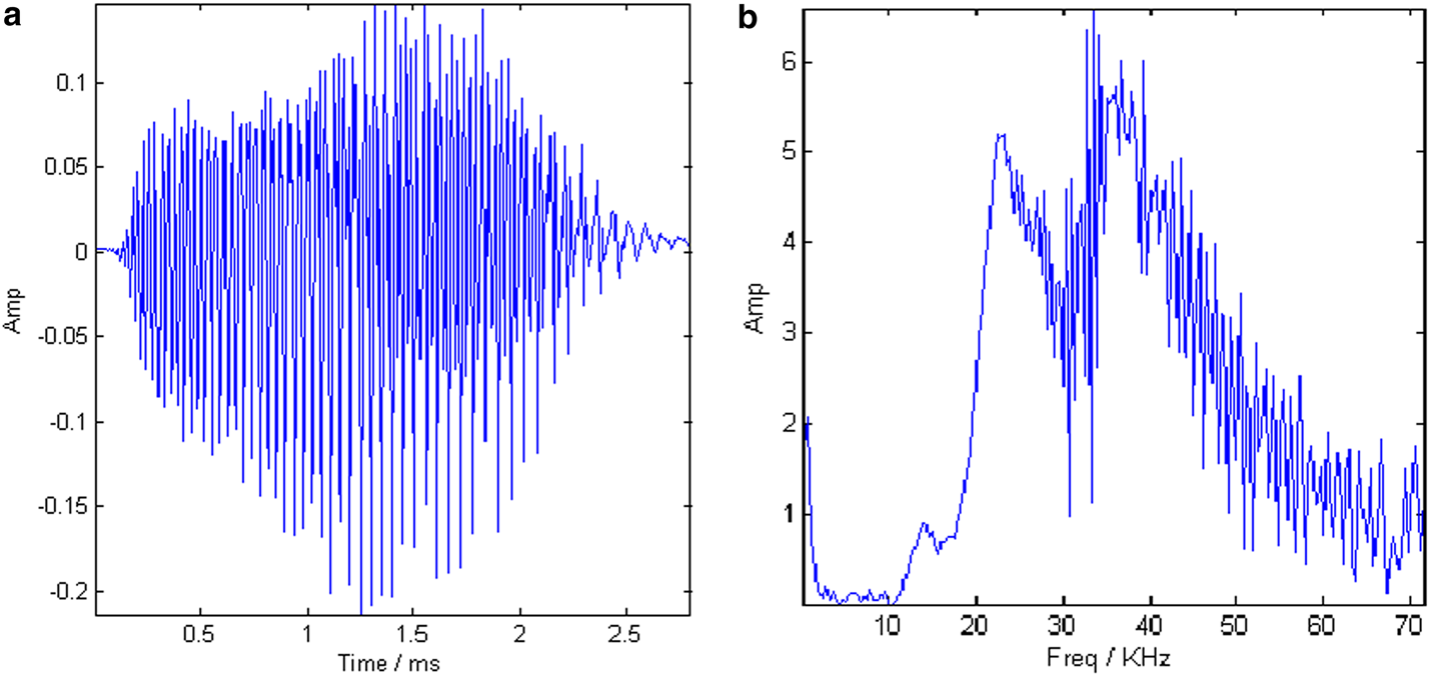
The waveform and spectrum of Bat echolocation signal. **a** Time waveform. **b** Spectrum

In the last experiment, PCFT-based method is employed to analyze a bat echolocation signal (http://dsp.rice.edu/software/bat-echolocation-chirp). The digitized echolocation signal is a nonlinear multicomponent FM signal. There are 400 samples and the sampling period is 7 μs. It can be observed in Fig. 13 that the signal lasts for about 2.5 ms and its energy distributes mainly from 20 kHz to 60 kHz. But the component composition can not be revealed in individual time or frequency domain.

**Fig. 14:**
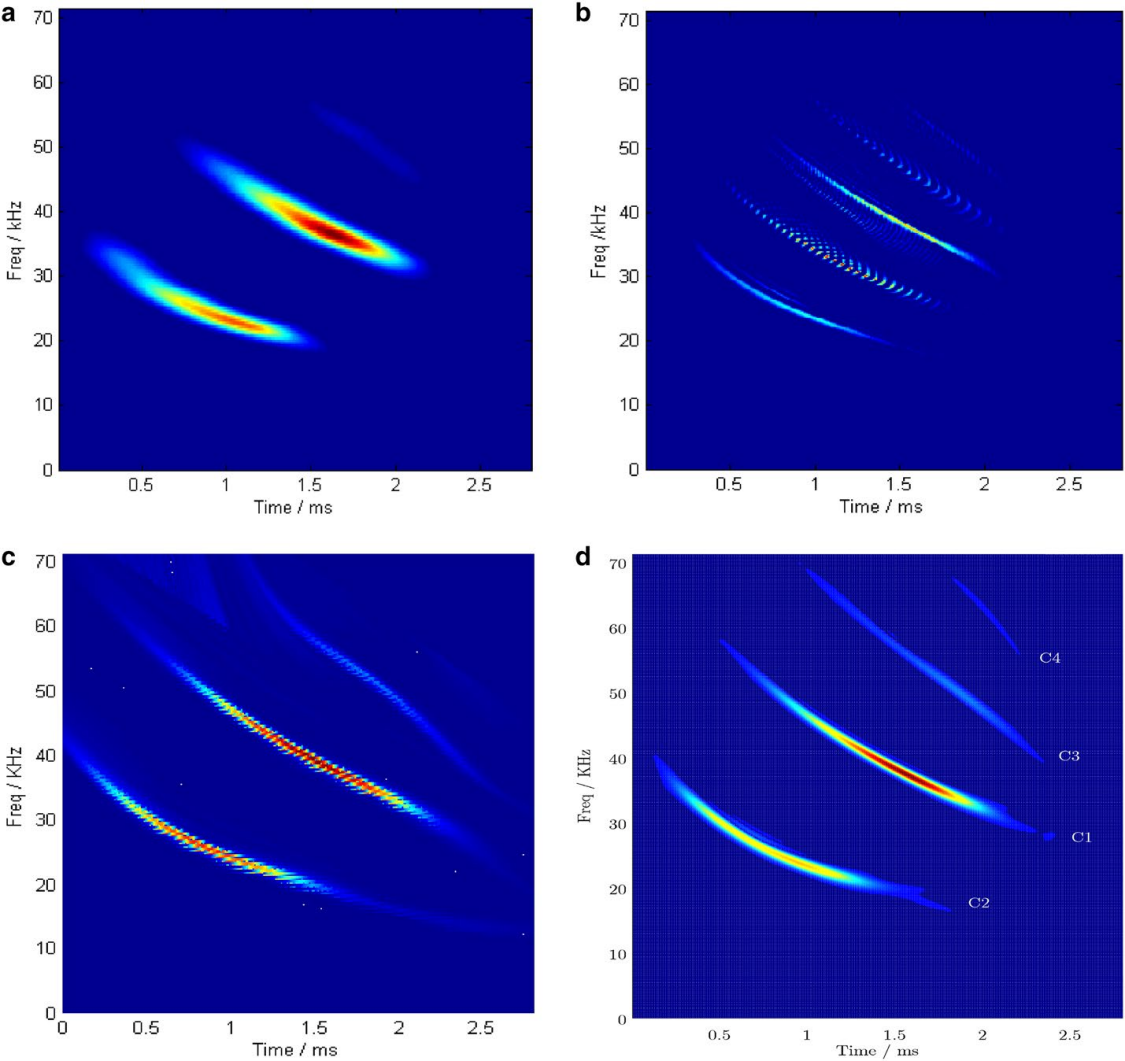
TFRs obtained by different methods. **a** STFT. **b** WVD. **c** Assembled PCT. **d** Assembled TFR of PCFT

Similarly, STFT, WVD and PCT are also taken into account for the sake of comparison. The TFRs achieved by these methods are depicted in Fig. 14. The parameters of PCFT are estimated by PSO algorithm and listed in Table 3. The STFT can only obtain blurred time–frequency signatures of strong components, as shown in Fig. 14a. For weak components, time–frquency ridges are unconspicuous because of their distributed energy on time–frequency plane. In Fig. 14b, WVD reveals the best time–frequency concentration for auto-terms, whereas cross-terms intefere and even distroy the weak components. Comparatively, it can be observed from Fig. 14c that the PCT method acquires a more concentrated ridge of every component by an iterative procedure including ridge extraction, IF approaching and component separation, yet weak components can not be detected due to their limited energy within the time window. In Fig. 14d, the assembled TFR obtained by PCFT-based method reveals both strong and weak components. Besides strong components, weak components are also detected and extracted with PCFT by energy accumulation.

**Table 3:** Estimated parameters of the bat echolocation signal

Component	Component 1	Component 2	Component 3	Component 4
$\tilde{\alpha }_{1}$	−629.60	−614.35	−498.14	1.3144e+3
$\tilde{\alpha }_{2}$	6.9769e+4	1.3417e+5	6.7696e+3	−3.2871e+5

Furthermore, we also consider the WVD results after tunable-Q wavelet transform (TQWT) on the real-world signal in (Pachorin and Nishad 2016) for better comparison, where the bat echolocation signal is processed at different SNR levels and with different threshold values. The TQWT followed by WVD can be regarded as an improvement of the traditional WVD on cross-term reduction. The method achieved good performance compared with conventional WVD, which, however, is at a sacrifice of time–frequency concentration. Moreover, decomposition of the signal with sub-bands leads to a broken time–frequency energy ridge. In contrast, the proposed method search global optimal parameters for PCFT and perform better in time–frequency concentration and continuous IFs for extracted components. Besides, from a perspective of energy accumulation, the PCFT can also detect the components in low SNR because of its global transform attribute.

## Conclusion

The main contribution in this paper is to put forward a PCFT. The transform integrates advantages both of FT as a global transform and PCT with a signal-dependent kernel, and accumulates component energy by a nonlinear way. With the optimal parameters, the PCFT converts the analyzed signal from time domain into a polynomial chirping Fourier domain, which can obtain concentrated energy of the interested component with distributed noise and other components. Moreover, an iterative procedure, including parameter estimation, PCFT, filter and recovery, is introduced to extract components from a noisy multicomponent signal. Simulations and experiments indicate that the proposed method can not only perform well in low-SNR environment, but also provide more time–frequency details.

Besides above advantages, the proposed method is also confronted with some problems in real applications. Just like PCT, the PCFT is suitable to analyze the signal with nonlinear IF. However, for the signal with highly oscillating IF, the method cannot guarantee sufficient estimation accuracy of the IF due to Runge phenomenon. Moreover, in real application, IF approximation error and improper filter usually cause large residual energy of the extracted component after filtering. The residual energy is typically considered as interference in later component extraction step. This will be even worse for weak components.

Therefore, the improvement of the current PCFT for better IF estimation will be a direction in the future. Our next study will focus on kernel selection of the parameterized chirping Fourier transform for signals with various IFs. Thus, a suitable kernel for the analyzed signal can accumulated component energy in the new transform domain, resulting in better IF approximation and component extraction. Moreover, adaption of the proposed method under different color noise will also be studied for multicomponent non-stationary signals in more applications.

## Abbreviations

PCFT: polynomial chirping Fourier transform
HHT: Hilbert Huang Transform
EMD: empirical mode decomposition
IMF: intrinsic mode functions
FB: Fourier–Bessel
FT: Fourier transform
IF: instantaneous frequency
NPTFA: non-parameterized time–frequency analysis
PTFA: parameterized time–frequency analysis
STFT: short-Time Fourier Transform
WT: wavelet Transform
WVD: Wigner-Ville Distribution
FM: frequency modulated
CT: chirplet transform
PCT: polynomial chirplet transform
SCT: spline chirplet transform
WBT: warblet transform
GWBT: generalized warblet transform
SNR: signal-to-noise ratio
TBD: track-before-detection
PSO: particle swarm optimization
TFR: time–frequency representation
NLFM: nonlinear frequency modulated
LFM: linear frequency modulated
TQWT: tunable-Q wavelet transform
